# Characterizing the Effect of Adding Boron Nitride Nanotubes on the Mechanical Properties of Electrospun Polymer Nanocomposite Microfibers Mesh

**DOI:** 10.3390/ma15051634

**Published:** 2022-02-22

**Authors:** Ohood Alsmairat, Nael Barakat

**Affiliations:** Department of Mechanical Engineering, The University of Texas at Tyler, 3900 University Blvd., Tyler, TX 75799, USA; nbarakat@uttyler.edu

**Keywords:** fiber, nanocomposite, mesh, electrospinning, BNNT

## Abstract

Electrospun fibrous meshes have a variety of applications such as filtration, drug delivery, energy storage, and engineered tissues due to their high surface area to mass ratio. Therefore, understanding the mechanical properties of these continuously evolving meshes is critical to expand and improve their performance. In this study, the effect of adding Boron Nitride Nanotube (BNNT) to Polymethylmethacrylate (PMMA) composite meshes on the mechanical properties of the polymer is studied. Electrospinning is used to fabricate microfiber meshes of PMMA and BNNT-PMMA. The fabricated meshes are tested experimentally with a uniaxial tensile tester. In addition, a theoretical model is introduced to investigate the effect of the number of fibers and the diameter of fiber inside the mesh on Young’s Modulus and Tensile Strength of the PMMA mesh. By adding 0.5% BNNT to the PMMA, Young’s Modulus and Tensile Strength of the PMMA mesh improved by 62.4% and 9.3%, respectively. Furthermore, simulated results show enhanced mesh properties when increasing the number of fibers and the single fiber diameter inside the mesh. The findings of this study help in understanding the mechanical properties of the nanocomposite electrospun meshes which expands and improves its utilization in different applications.

## 1. Introduction

Tendons, ligaments, and joint capsules’ injuries constitute almost half of the musculoskeletal injuries, which are rapidly increasing due to aging, sports’ practice, and other reasons [1]. The slow recuperation and rebuilding process of the damaged tissues can be assisted by fabricated bio-polymer engineered tissues or what is known as artificial organ tissues. However, appropriate strength and the side immune impermissible effects continue to impose limitations on the use of these tissues [2].

Recently, the employment of the electrospinning fabrication technique for fabricating synthetic nanostructured bio-materials has proven to be a very promising solution to overcome limitations associated with artificial organ tissues [3]. Since nanocomposites are produced by adding reinforcements into matrix materials, their properties can become outstanding by mixing the advantages of fibers and added particles, and hence presents a method to improve the properties of electrospun fibers [4]. As a result, they provide significant opportunities to optimize the performance of metal matrix composites for potential applications in different engineering and medical fields. In the last decade, different attempts have been made to enhance various mechanical properties of materials in the micro-and nanoscale range. This includes significant improvements in polymer properties achieved by adding nanotubes [5,6]. Superior mechanical properties can be obtained by growing carbon nanotubes (CNTs) on carbon fibers [6]. The tensile properties of hybrid composites were improved by increasing the glass fiber content [7]. Theoretical and experimental studies of the effective elastic modulus for multiphased hybrid composites provide an insightful understanding of various kinds of multiphase hybrid composites [8]. Experimental and theoretical predictions were in good agreement for the tensile strength and modulus of short and randomly oriented hybrid natural fiber composites [9]. Increasing the carbon content in glass–carbon fiber composites increases the stiffness and reduces the weight and strength compared to conventional glass fiber polymer composites [10]. Even though the fracture strain and the energy dissipated during the fracture were increased, the enhancement of adding glass fibers to the composite strength was not utilized because of the early fracture of the carbon fibers [11]. Multiscale composites revealed significant improvement in the elastic and storage modulus, strength, and impact resistance [12].

Due to the wide range of applications of nanofibers, different types of techniques were used in the production process, such as drawing [13], template synthesis [14], phase separation [15], selfassembly [16], and electrospinning [17,18,19,20,21]. Among all of these techniques, electrospinning is considered to be the most efficient. Electrospinning is a fiber production method that uses electric force to draw charged threads of polymer solutions or polymer melt up to fiber diameters in the order of some hundred nanometers. Electrospinning shares characteristics of both electrospraying and the conventional solution dry spinning of fibers. To produce the best electrospun fibers for the desired application, many parameters must be examined. The solution concentration, viscosity, type of solvents, and spinning voltage are considered to be important parameters that influence nanofiber production. Solutions with higher viscosity will have fewer beads formed in the produced fibers [22]. Increasing the concentration of the nanotubes can result in fibers with a larger diameter and fewer beads [23]. Using different solvents can also produce different types of nanofibers, such as ring-like fibers, beadlike fibers, and ultrafine and nanoporous fibers [23]. Increasing the spinning voltage produces irregularly shaped fibers at higher concentrations [23]. The mechanical properties of fibers are independent of the concentration of the solution used. These properties only depend on the features of the fibers, such as the smaller diameters and fewer beads [24]. Fibers produced with smaller diameters will have better mechanical properties, such as a higher Young’s modulus, better strength, and more toughness [25,26,27,28]. With the electrospinning technique, fiber properties such as the diameter, alignment, degree of fusion, and porosity can be controlled and enhanced [24]. Such a technique has been used widely in tissue engineering for biomedical applications, such as tendons, ligaments, cartilages, intervertebral discs, and blood vessels [3,29,30,31]. Moreover, the fabricated tissues will possess a uniform and homogeneous structure, and their mechanical stiffness controls the tissue regeneration process under cyclic and continuous stresses [32,33]. Therefore, investigating the mechanical behavior of the electrospun mesh created from nanocomposite fibers is highly needed to develop functional scaffolding technology.

The most important structural properties for the fiber mesh consist of the fiber diameter and the degree of alignment of constituent fibers. During electrospinning, fusion among fibers may occur due to removing the residual solvents after fiber deposition, or can be manually introduced to obtain fibrous membranes. Such fusion can affect the mechanical properties of fiber meshes [34]. On the one hand, an increase in fusion among fibers in the random mesh increases the strength of the mesh [35]. However, any small crack in the fiber will lead to a mesh failure, which lowers the toughness of fiber meshes. This proves the importance of considering fusion while characterizing the mechanical properties of the fibers. On the other hand, the alignment of the nanotubes in the fibers plays a significant role in cell adhesion and migration and increases the fiber meshes’ strength. Ignoring the degree of the alignment effect while studying mechanical properties can lead to inaccurate results. A multiscale model based on a computational technique shows that increasing the diameter or alignment of fibers enhances the tensile strength of fiber meshes [36]. However, the estimated results do not agree with experimental results due to some simplifications related to the homogeneity, free rotation allowance, and constant degree of fiber crosslinking. A similar study with finite element modeling shows similar outcomes [37]. Furthermore, mathematical modeling has been used and validated experimentally to investigate the length, diameter, and alignment of fibers, and shows that, for non-bonded fibrous material, the mechanical behavior varies from their bulk material [38,39]. Experimentally, it was found that the orientation of the fibers in the fiber mesh plays a significant role in the strength of the mesh [40], the direction of applied load [41], and the bulk mechanical properties of the polymer used for generating fiber meshes [42]. However, the above studies could not capture the evolution of microstructural changes in the fiber meshes on being stressed by the tensile force. Therefore, it is essential to understand the mechanical behavior of nanocomposite fiber meshes during tensile testing for tissue engineering applications.

In this study, an electrospun fiber mesh made from boron nitride nanotubes (BNNT) added to a polymer matrix of PMMA is fabricated and experimentally characterized. In parallel, a theoretical study of the mechanics of fiber meshes is introduced and validated with the experimental findings. The mechanical behavior and properties of the meshes were investigated experimentally dependent on the tensile strength. The results illustrated the transition of random curved fibers to straight fibers in a mesh during tensile testing. One of the results of this study emphasized the effect of the number and the diameter of the fiber inside the mesh on Young’s modulus. Section 2 of this study includes a discussion of the experimental setup and materials used to carry out the study. In Section 3, the theoretical model is developed using Cauchy stress, followed by a discussion of the results in Section 4. Finally, conclusions are drawn in Section 5.

## 2. Setup and Materials

**Figure 1:**
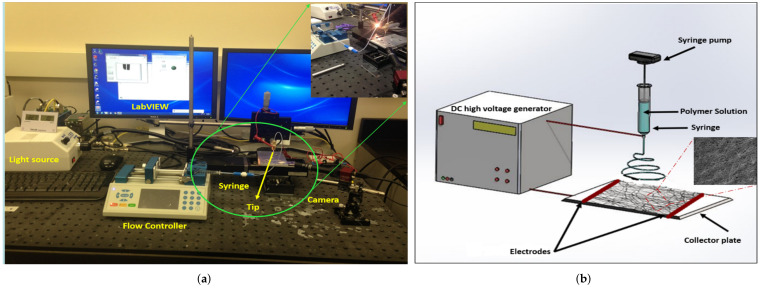
Electrospinning experimental setup: (**a**) real setup, (**b**) schematic.

The experimental apparatus for the electrospinning setup is shown in Figure 1a. The setup used fabricated nanocomposite fiber mesh polymers. The setup consisted of a high voltage power supply source, a camera with a high-resolution lens, a tip (needle), a flow controller pump, and a light source. The LabVIEW 2019 software was used to control fiber production with this technique. Furthermore, a schematic diagram of the electrospinning experimental setup used for the nanocomposite fiber mesh production is shown in Figure 1b. During the electrospinning process, a high voltage is applied to a needle that contains a polymer solution to create an electric field between the tip and a substrate screen, as shown in Figure 1b. The voltage source includes two electrodes, one of which is in contact with the polymer solution, whereas the second is in contact with the substrate screen. This setup creates an electrostatic force between the two electrodes. With a large enough voltage, a higher electrostatic force produced opposite the droplet surface tension results in a conical-shaped droplet known as a Taylor cone. The solution is ejected from the tip toward the substrate screen when the electrostatic force exceeds the surface tension force. After the ejection, the solvent evaporates, and microfibers accumulate on the substrate.

**Figure 2:**
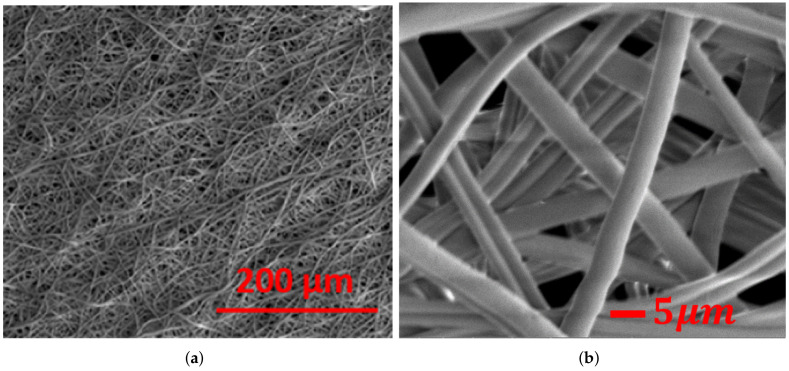
SEM images of electrospun BNNT-PMMA nanocomposite: (**a**) fiber mesh (**b**) zoomed in on the fiber mesh.

Acetone solvent and PMMA solute (50,000 in molecular weight, purchased from Sigma-Aldrich) were used to prepare the polymer solution needed for nanocomposite fiber mesh production. The PMMA was dissolved in acetone to prepare a 23% of PMMA-acetone polymer solution. The boron nitride nanotube (BNNT) solution was synthesized using the high-temperature pressure method and purchased from BNNT LLC. Then, BNNT was dispersed in dimethylformamide (DMF) using ultrasonication for several hours. After that, the prepared (BNNT) solution was mixed with the PMMA solution to make a polymer of BNNT-PMMA with 0.5% BNNT-weight concentration. Using the electrospinning setup shown in Figure 1, we set the parameters as follows: First, the spinning voltage was selected to be 9 kV. The selection of this voltage was based on extensive trials to find the best voltage that can create smooth fibers with uniform diameters and out of beads. In addition, it is essential for high viscous solutions. Furthermore, using a very high voltage will make the production process nonuniform and give fibers with a rough surface, whereas using a low voltage will not help to produce a fiber. Second, the flow rate was controlled to stay at roughly (10 μL/min) for the continuity of the production process of the microfiber from the tip. Third, the glass nozzle diameter was selected to be within a 5–20 μm range to prevent the solution from being jammed. Finally, an optimal distance of 3–4 cm between the two electrodes was selected to help in evaporating the solvent after ejections from the Taylor cone. Using these settings, randomly electrospun fiber meshes from PMMA and BNNT-PMMA solutions were fabricated at room temperature. The fabricated meshes were removed from the electrodes and placed on a tray in the oven for four hours to remove any remaining solvent from the mesh. An SEM image for a sample mesh fabricated from BNNT-PMMA solution is shown in Figure 2a. The average diameter of the single fiber was 5.7 ± 0.79 μm. Zooming in on the mesh, we can see that the fibers are homogeneous and have constant cross-section area in random orientation, as shown in Figure 2b.

**Figure 3:**
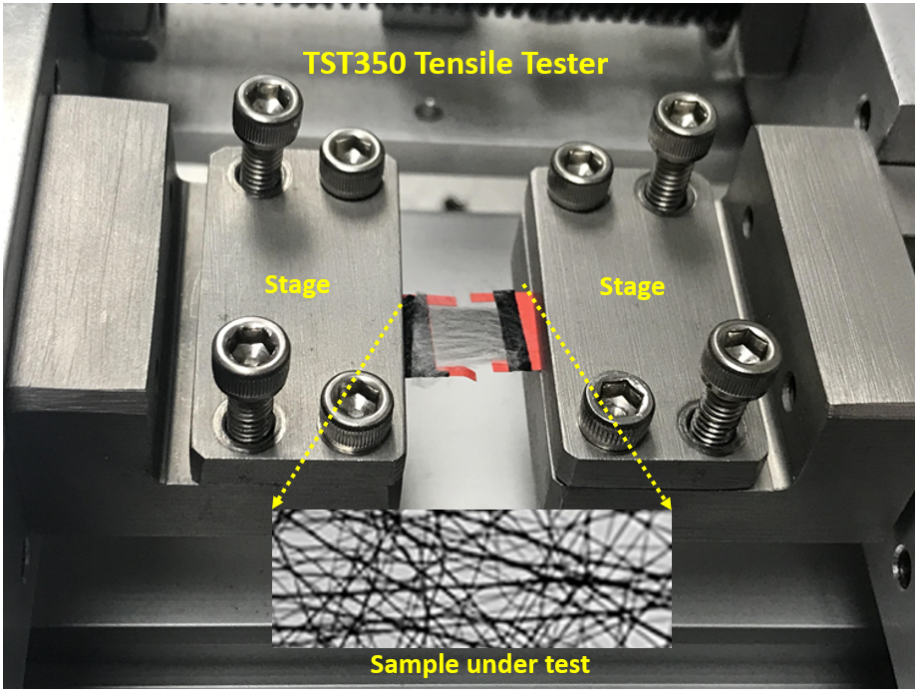
The uniaxial tensile tester experimental setup, with a sample under testing.

The fabricated meshes were tested using a TST350 tensile tester from Linkam Scientific Instrument (Tadworth, UK), Figure 3, to extract its tensile strength properties. The fabricated mesh with random fiber orientation was attached to the tensile tester stage at the gap between the clamped screws. The clamped screws were used to control the horizontal and vertical alignment of the fiber mesh. An axial tensile load of 2.5 N was applied to the fabricated mesh at a rate of 1 μm/s. A series of tensile tests were performed on each type of the fabricated fibers meshes. While the load was applied, the displacement in the fabricated fiber mesh was recorded, and the strain in each mesh was calculated. After that, a stress–strain curve could be generated for each sample. A sample of the fabricated mesh under testing is shown in Figure 3.

## 3. Theoretical Model

Following the procedures in Pai et al. [43], a theoretical model for two-dimensional fibers mesh can be extracted by using the first derivative of the Cauchy stress to calculate the Young’s modulus as follows:

(1) $$E_{mesh}=\frac{1}{t_{mesh}}\langle \frac{\lambda_{1}\partial T_{1}}{\partial \lambda_{1}}{|}_{\lambda_{1}=1}\rangle =\frac{\upsilon k_{f}r_{0}^{2}sin^{2}\theta_{0}}{t_{mesh}}$$

where $E_{mesh}$ is the Young’s modulus of the mesh, $t_{mesh}$ represents the thickness of the mesh, $\lambda_{1}$ is the principal stress with the plane, $T_{1}$ is the Cauchy stress tensor, $r_{0}$ is the original fiber length, and $\upsilon =\frac{n}{a}$, where *n* is the number of fibers, *a* is the area of the mesh, and where $k_{f}$ is the fiber axial stiffness in units of force per unit length. In addition, the term $sin^{2}\theta_{0}$ is the average two-dimensional ensemble, which is taken to be equal to 1/2 for randomly oriented fibers. Hence, the previous equation is simplified to be:

(2) $$E_{mesh}=\frac{\upsilon k_{f}r_{0}^{2}}{2t_{mesh}}$$

The relation between the $k_{f}$ and the Young’s modulus of a single fiber ($E_{f}$) is given by [43]:

(3) $$k_{f}=\frac{E_{f}A_{f}}{r_{o}}=\frac{E_{f}\left(\frac{\pi d^{2}}{4}\right)}{r_{o}}$$

where $A_{f}$ is the cross-section area of a single fiber and *d* is the diameter of the single fiber. Using Equation (3), we can calculate the theoretical Young’s modulus for the PMMA mesh. Toward this, Equation (3) is substituted for Equation (2), and the final form for the Young’s modulus for the PMMA mesh as a function of the diameter of the single fiber will be:

(4) $$E_{mesh}=\frac{\pi \upsilon E_{f}r_{0}}{8t_{mesh}}d^{2}$$

## 4. Results and Discussions

**Figure 4:**
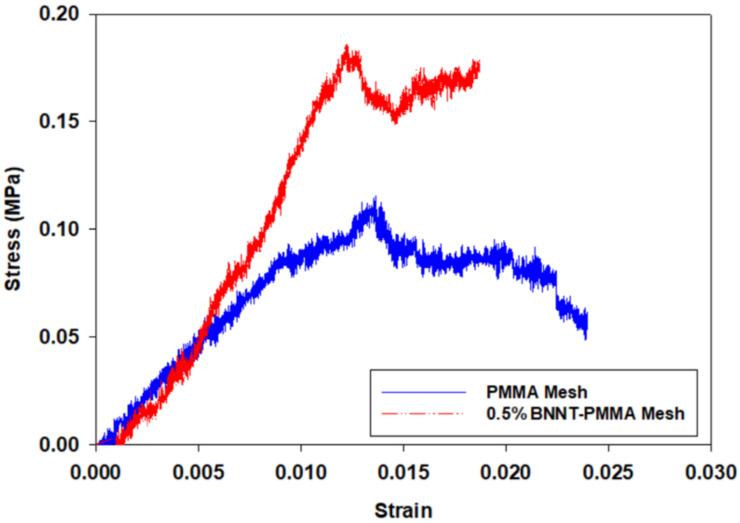
Sample of stress–strain curve extracted from the tensile tester for one mesh from PMMA and BNNT-PMMA fiber.

**Figure 5:**
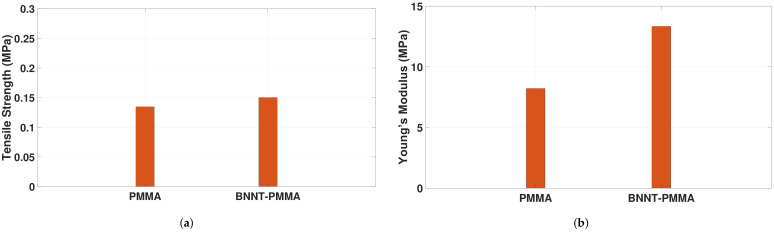
Tensile strength properties enhancement in BNNT-PMMA nanocomposite: (**a**) the average tensile strength; (**b**) the average Young’s modulus.

Nanocomposites-based meshes were fabricated and tested using the experimental setup based on the TST350 tensile tester presented in Figure 3. Stress-strain curves were extracted for six samples of the PMMA and BNNT-PMMA fiber meshes. The average thickness of the tested meshes was 100 μm, and all tests were performed at room temperature after the solvent was fully evaporated in an oven. In addition to the evaporation of the solvent, the use of the oven can significantly increase the bonding between fibers to reduce the effect of fiber sliding during the tensile test. Figure 4 shows the stress–strain curve for one of the six mesh samples fabricated from both PMMA and BNNT-PMMA solutions at a 0.5% concentration. When the tensile test started, the cross-section of the mesh started to decrease due to the reorientation of the curved fiber to be aligned with the direction of the force applied. After that, the stress started to increase until it reached its maximum values, then dropped due to sliding of the fibers and continued to reach the fracture point where all fibers inside the mesh were fractured. Comparing the tensile strength properties extracted from the PMMA and BNNT-PMMA stress-strain curves in Figure 4, mechanical strength seems to be significantly improved by adding the BNNT nanotubes. Adding BNNT to the PMMA enhanced both the tensile strength and Young’s Modulus where both increased from from (0.1346 ± 0.007 and 8.21 ± 0.568) MPa for the PMMA to (0.15 ± 0.02 and 13.33 ± 0.49) MPa for the BNNT-PMMA, respectively. The average tensile strength (σ) and the Young’s modulus (*E*) from the six samples of fiber meshes for each type are summarized in Figure 5 and also listed in Table 1. The average mechanical strength of the BNNT-PMMA mesh was significantly improved with the addition of the BNNT at a concentration of 0.5% wt%.

**Figure 6:**
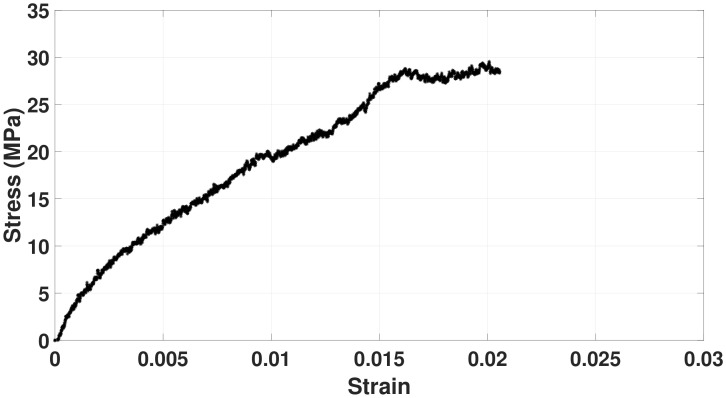
Sample of stress–strain curve extracted from the tensile tester for a single BNNT-PMMA fiber to extract the Young’s modulus of a single fiber ($E_{f}$).

**Figure 7:**
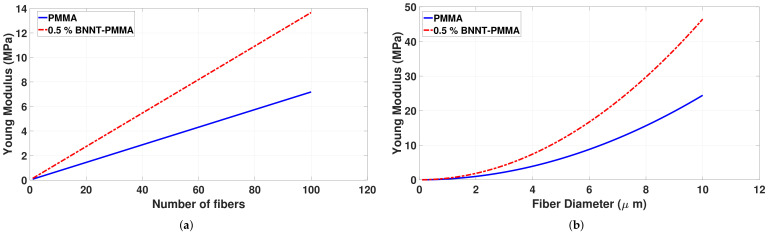
The variation of the Young’s modulus of the PMMA and BNNT-PMMA nanocomposite mesh with: (**a**) number of the single fibers; (**b**) diameter of the single fibers.

The effect of the number and diameter of fibers inside the mesh on Young’s modulus was investigated using a theoretical model. Toward this, the stress-strain curve for a single BNNT-PMMA fiber was extracted experimentally to find the Young’s Modulus of a single fiber ($E_{f}$). Figure 6 shows the stress-strain curve for a single BNNT-PMMA fiber sample. The slope of the linear portion of this curve is calculated, and the average Young’s Modulus of a single fiber ($E_{f}$) was found to be 2.23 GPa. Young’s Modulus for PMMA single fiber was taken to be 1.28 GPa, [44]. Figure 7a shows the variation of the Young’s Modulus with the variation of the number of fibers inside the mesh, where a directly proportional relation seems to exist. Increasing the number of fibers linearly increases the Young’s Modulus of the mesh. This increment is due to the increased number of bonded fibers inside the mesh, making it stronger. Studying the effects of fiber diameter which are randomly oriented inside the mesh on the young modulus reveled that increasing the diameter of the single fiber inside a mesh causes an increase in the Young’s Modulus as shown in Figure 7b. Using the theoretical model in Equation 3, the theoretical value of Young’s modulus for the PMMA mesh was calculated and found to be 8.35 MPa. Comparing this value to the experimental value shown in Figure 5b, it turns out that they are almost identical.

## 5. Conclusions

Experimentally electrospun fiber mesh made of a polymer matrix of PMMA and infused with BNNT nanotubes was fabricated in the laboratory. The mechanical properties of this mesh were experimentally tested to extract the stress-strain curves using a tensile tester. Moreover, a theoretical model to estimate the mechanics of fiber meshes as a function of the number and diameter of fibers was developed and utilized to calculate Young’s modulus of the tested mesh. Results from the theoretical model and laboratory tests were found to be in good agreement. The tensile strength properties of the PMMA polymer were enhanced significantly by infusing BNNTs nanotubes in the mesh. Moreover, the theoretical model has shown that Young’s Modulus can be increased by increasing the number of fibers and fiber diameter inside the mesh. Eventually, the study emphasizes the significance of adding the BNNT to the PMMA matrix as it improves the tensile strength properties of electrospun fiber meshes. It projects them as essential design criteria while evaluating a fibrous material for load-bearing engineering tissues.

## Figures and Tables

**Table 1:** The average tensile strength properties of the PMMA and BNNT-PMMA fiber mesh.

Mesh	Young’s Modulus (MPa)	Tensile Strength (MPa)
PMMA	8.21 ± 0.568	0.1346 ± 0.007
0.5% BNNT	13.33 ± 0.49	0.15 ± 0.02

## Data Availability

Not applicable.
